# Protective effects of (-)-epigallocatechin-3-gallate against acetaminophen-induced liver injury in rats)

**DOI:** 10.7603/s40681-015-0015-8

**Published:** 2015-08-12

**Authors:** Hsien-Tsung Yao, Yu-Chi Yang, Chen-Hui Chang, Hui-Ting Yang, Mei-Chin Yin

**Affiliations:** Department of Nutrition, China Medical University, No. 91, Hsueh-Shih Road, 404 Taichung, Taiwan

**Keywords:** (-)-Epigallocatechin-3, gallate;, Acetaminophen; Cytochrome P450; Hepatotoxicity;, Rats

## Abstract

(-)-Epigallocatechin-3-gallate (EGCG) is the most abundant catechin with various biological activities found in tea. In this study, the effects of EGCG on the metabolism and toxicity of acetaminophen in rat liver were investigated. Male Sprague-Dawley rats were fed a controlled diet without or with EGCG (0.54 %, w/w) for 1 week and were then intraperitoneally injected with acetaminophen (1 g/kg body weight) and killed after 12 h. Concentrations of acetaminophen and its conjugates in plasma and liver were then determined. The cytochrome P450 (CYP) and phase II enzymes activities were also evaluated. Rats fed the EGCG diet had lower plasma alanine aminotransferase and aspartate aminotransferase activities, as indices of hepatotoxicity, after acetaminophen treatment. Morphological damage by acetaminophen was lower in rats fed the EGCG diet. In addition, EGCG significantly reduced hepatic activities of midazolam 1-hydroxylation (CYP3A), nitrophenol 6-hydroxylase (CYP2E1), UDP-glucurosyltransferase, and sulfotransferase. Finally, EGCG feeding reduced acetaminophen-glucuronate and acetaminophen-glutathione contents in plasma and liver. These results indicate that EGCG feeding may reduce the metabolism and toxicity of acetaminophen in rats.

## Introduction

Phytochemicals are found in plant-based foods such as fruits, vegetables, beans, and grains, and they may reduce the risk of a number of chronic diseases including cancer, cardiovascular disease, and diabetes [1]. It is known that phytochemicals can also influence the pharmacological activity of drugs and their toxicities by modifying the drug metabolism system, including drug-metabolizing enzymes and transporters [2, 3].

Acetaminophen (N-acetyl-*p*-aminophenol, APAP) is an antipyretic and analgesic drug. When an overdose is taken, it can induce severe hepatotoxicity in both humans and experimental animals [4]. APAP is primarily metabolized in the liver by phase II conjugating enzymes, mainly UDP-glucurosyltransferase (UGT) and sulfotransferase (ST), to generate the nontoxic metabolites APAP-glucuronate and APAP-sulfate [4]. The initiation of APAP-induced liver injury results from the cytochrome P450 (CYP)-mediated metabolism of APAP into a reactive metabolite, *A*-acetyl-*p*-benzoquinone imine (NAPQI), which exerts its toxicity by covalently binding to cellular macromolecules such as proteins, lipids, and DNA [5]. NAPQI also reacts with glutathione (GSH), leading to cellular GSH depletion and the production of reactive oxygen species in the liver. Studies have shown that natural products that decrease CYP enzyme activity, increase antioxidant enzyme activity or GSH levels may attenuate APAP-induced liver toxicity [6, 7].

(-)-Epigallocatechin-3-gallate (EGCG) is the most abundant and active polyphenol in green tea. Studies suggest that EGCG reduces the development and progress of various diseases such as cancer and cardiovascular disease [8, 9]. The principal hypothesis associated with the putative benefits of tea polyphenols or EGCG is linked to its strong free radical scavenging and antioxidant and anti-inflammation properties, as well as its modulating effects on drug-metabolizing enzymes, which reduce the bioactivation of carcinogens [10, 11]. Studies have shown that EGCG reduces hepatic CYP3A activity and increases the oral bioavailability of nicardipine and diltiazem in rats [12, 13]. Also, in vitro studies have indicated that EGCG reduces UGT and SUT activities [14, 15]. However, there is currently a lack of information about the effect of EGCG on the phase II detoxifying enzymes in vivo.

Recently, EGCG has been shown to have hepatoprotective activity against chemically induced liver injuries [16, 17]. However, the mechanism of action remains unknown. In this study, we investigated whether EGCG feeding could change the metabolism and toxicity of APAP in rats.

## 2. Materials and methods

### 2.1 Materials

Acetaminophen, methoxyresorufm, resorufin, *p*-nitrophenol, 4-nitrocatechol, NADPH, glutathione, 1-chloro-2,4-dinitrobenzene, and heparin, were obtained from Sigma (St. Louis, MO, USA). Midazolam and 1-hydroxymidazolam were purchased from Ultrafine Chemicals (Manchester, UK). All other chemicals and reagents were of analytical grade and were obtained commercially. EGCG was purchased from Huzhou Ruzhou Rongkai Foliage Extract Co. LTD (Huzhou, China). The purity of the EGCG used was > 99 % as determined by high performance liquid chromatography (HPLC).

### 2.2 Animal study

First, in the preliminary study, we investigated the effect of EGCG on the drug-metabolizing enzymes in rat livers. Male Sprague-Dawley rats (aged 6 weeks) obtained from BioLASCO in Ilan, Taiwan were used. Rats were fed a laboratory chow diet with or without 0.15 % and 0.54 % of EGCG for 1 week. Second, to investigate the effect of EGCG on the metabolism and toxicity of APAP, the male Sprague-Dawley rats weighing 210± 10 g (6 weeks old) were randomly divided into three groups with six rats in each group. The animals in Group 1 (control group) and 2 were fed a laboratory chow diet. The animals in Group 3 were fed the same diet fortified with 0.54 % EGCG. The daily dose of EGCG was about 460 mg/kg in rats, which was equivalent to the dose used in a previous study that found that EGCG did not change the liver function [18]. The rats were all housed in plastic cages in a room kept at 23± 1°C with 60± 5 % relative humidity and a 12-h light-dark cycle. Food and drinking water were available *ad libitum* for 1 week. At the end of the 1-week feeding period, food was withdrawn for 12 h and a single 1000-mg/kg dose of APAP, as a solution in polyethylene glycol 400/water (50/50, v/v), was intraperitoneally injected into each animal in Group 2 and 3. At 12 h after the APAP dose, the animals in all three groups were killed by exsanguination via the abdominal aorta while under carbon dioxide (70:30, CO_2_/O_2_) anesthesia. Heparin was used as the anticoagulant, and the plasma was separated from the blood by centrifugation (1750 x g) at 4°C for 20 min. Plasma alanine aminotransferase (ALT) and aspartate aminotransferase (AST) activity were measured immediately by use of commercial kits (Randox Laboratories, Antrum, UK). The liver samples of the three groups were excised and fixed in 10 % neutral formalin followed by dehydration in ascending grades of alcohol, clearing in xylene, and embedding in paraffin wax. Liver sections (5 μm thickness) were stained with hematoxylin and eosin (H&E) for the histological examination [19]. The other liver samples from each animal were stored at -80°C. Microsome preparation and enzyme assays were performed within 2 weeks of liver collection. The separated plasma was used for the determination of APAP, APAP-sulfate, APAP-glucuronate, and APAP-glutathione concentrations. This study was approved by the Animal Center Management Committee of China Medical University. The animals were maintained in accordance with the guidelines for the care and use of laboratory animals as issued by the Animal Center of the Ministry of Science and Techonology, Taiwan.

### 2.3 Determination of APAP and APAP conjugates in plasma and liver

Liver homogenates were prepared by homogenizing each gram of liver with 4 mL of ice-cold phosphate-buffered saline (pH 7.4). For determining APAP, APAP-sulfate, and APAP-glucuronate, plasma samples and liver homogenates were diluted 10-fold with control plasma and control liver homogenates, respectively. For determining APAP-glutathione, plasma samples and liver homogenates were not diluted. An aliquot (50 μL) of plasma or liver homogenate was then extracted with 100 μL of acetonitrile and centrifuged at 10,000 x *g* for 15 min at 4°C. The acetonitrile extract thusly obtained was then analyzed by an HPLC-mass spectrometry (HPLC/MS) method. Calibration standards of APAP, APAP-sulfate, APAP-glucuronate, and APAP-glutathione were prepared by serial dilution of the stock solution of each compound with control plasma or liver homogenate yielding final concentrations of APAP, APAP-sulfate, APAP-glucuronate, or APAP-glutathione that ranged from 1 to 200 pg/mL of plasma or liver homogenate. An aliquot (50 μL) of the spiked plasma or liver homogenate was then extracted with 100 μL of acetonitrile as described above.

To determine hepatic APAP protein adducts, liver homogenate was filtered through Nanosep centrifugal devices (Pall Life Sciences, Ann Arbor, MI, USA) with a membrane molecular weight cutoff of 30 kDa to remove low molecular weight compounds with the potential to interfere in the assay. The filtrate was then digested for 16 hours with proteases to free the APAP-cysteine from APAP protein adducts [7].

The HPLC/MS system consisted of an Agilent 1100 series LC System (Palo Alto, CA, USA). A Mightysil RP-18 GP column (5 μm, 250 × 4.6 mm i.d., Kanto Chemical) was used for the determination of APAP. An Agilent Zorbax Eclipase XDB-C18 column (5 μm, 250 × 3.0 mm i.d., Agilent) was used for the determination of APAP conjugates. The HPLC system was interfaced to an Agilent MSD mass spectrometer equipped with an electrospray ionization source. The column temperature was set to 25°C. Mobile phase A was 10 mM ammonium acetate containing 0.5 % formic acid. Mobile phase B was acetonitrile containing 0.5 % formic acid. An isocratic system containing 20 %A/80 %B was used to determine APAP. The flow rate was 0.5 mL/min. The retention time of APAP was 5.0 min. A gradient system with the following composition was used to determine the APAP conjugates: 90 % A (0-2 min), 90 % A to 10 % A (2-3 min), 10 % A (3-5 min), 10 % A to 90 % A (5-6 min), 90 % A (6-12 min). The retention times of the analytes were 2.5 min (APAP-glucuronate), 2.4 min (APAP-sulfate), 2.5 min (APAP-glutathione), and 2.7 min (APAP-cysteine), respectively. The flow rate was 0.5 mL/min. The injection volume was 10 μL. The MS data acquisition was via selected ion monitoring. Ions representing the positive of the testing compound were selected and the peak was measured.

### 2.4 Preparation of liver microsomes

The frozen liver was thawed and then homogenized (1:4, w/v) in an ice-cold 0.1 M phosphate buffer (pH 7.4) containing 1 mM ethylenediaminetetraacetic acid (EDTA). The homogenates were centrifuged at 10,000 x *g* for 15 min at 4°C. The supernatants were then centrifuged at 105,000 x *g* for 60 min. The resulting microsomal pellets were suspended in a 0.25 M sucrose solution containing 1 mM EDTA and were stored at -80°C until they were used. The microsomal protein concentration was determined by using a BCA protein assay kit (Pierce, Rockford, IL, USA).

### 2.5 Drug-metabolizing enzyme activity assays

The CYP enzyme activities were determined by the previously reported HPLC/MS methods [20]. Methoxyresorufin (5 μM), *p*-nitrophenol (50 μM), and midazolam (2.5 μM) were respectively used as the probe substrates for methoxyresorufin *O*-demethylation (CYP1A2), *p*-nitrophenol 6-hydroxylation (CYP2E1), and midazolam 1-hydroxylation (CYP3A). 0.2 mg/ml of microsomal protein concentrations and 15 min of incubation times were used for the determinations of CYP1A2 and CYP2E1. For determining CYP3A activity, 0.2 mg/ml of microsomal protein concentrations and 5 min of incubation times were used. Enzyme activities were expressed as pmol of metabolite formation/min/mg protein.

**Table 1:** – Drug-metabolizing enzymes in the liver of rats fed the EGCG diet for 1 week.

	Control	EGCG 0.18 %	EGCG 0.54 %
Testosterone 6Β-hydroxylase (CYP3A) (pmol/min/mg protein)	798.9± 43.8	599.7± 121.6*	452.2± 109.4*
*ρ*p-Nitrophenol 6-hydroxylase (CYP2E1) (pmol/min/mg protein)	325.5± 27.1	371.0± 34.0	357.3± 16.6
Methoxyresorufin O-demethylase (CYP1A2) (pmol/min/mg protein)	31.0± 4.5	34.0± 5.6	29.5± 4.5
UDP-glucurosyltransferase (nmol/min/mg protein)	53.9± 22.2	34.0± 3.4	24.5± 4.3*
Sulfotransferase (pmol/min/mg protein)	1,575.6± 47.5	1,386.0± 26.0*	1,397.1± 38.6*
Glutathione S-transferase (nmol/min/mg protein)	148.2± 4.4	108.9± 3.5*	116.4± 10.1*

Results are expressed as the mean± S.D. of three rats in each dietary group. *Significantly different from control group, *P* < 0.05.

The microsomal UGT activity was determined by using *p*-nitrophenol as the substrate where the rate of formation of *p*-nitrophenol glucuronic acid was measured by HPLC/MS [21]. The cytosolic ST activity was determined by using phosphoadenosine 5-phosphosulphate as the substrate and *p*-nitrophenol as the acceptor of the sulfate, and the rate of formation of adenosine 3,5-diphosphate was measured by HPLC/MS [22].

### 2.6 Determination of GSH and glutathione S-transferase (GST) activity

Liver homogenate was prepared by homogenizing each gram of liver with 10 ml of ice-cold 1.15 % KCl and centrifuging the homogenate at 10,000 x *g* for 15 min at 4°C. The resulting supernatant was used to determine the GSH content and GST activity. he GSH content in liver homogenates was determined by HPLC/MS [23]. GST activity was determined spectrophotometrically [24]. The protein concentration in tissue homogenates was determined by using a BCA protein assay kit (Pierce, Rockford, IL, USA).

### 2.7 Statistical analysis

Statistical differences among groups were calculated by using a one-way ANOVA (SAS Institute, Cary, NC, USA). The differences were considered to be significant at *P <* 0.05 as determined by independent-sample *t*-tests.

## 3. Results

Table 1 shows the effect of EGCG feeding on drug-metabolizing enzyme activity in the liver. Rats fed on the 0.18 % and 0.54 % EGCG diets for 1 week had significantly reduced *(P* < 0.05) testosterone 6Β-hydroxylase (CYP3A) in their livers. In addition, lower ST and GST activities was found in rats that were fed the EGCG diets (*P* < 0.05). The UGT activity was reduced only in 0.54 % EGCG group (*P* < 0.05). No significant difference in plasma ALT and AST activities was observed, indicating EGCG caused no hepatotoxicity (data not shown).

**Fig. 1:**
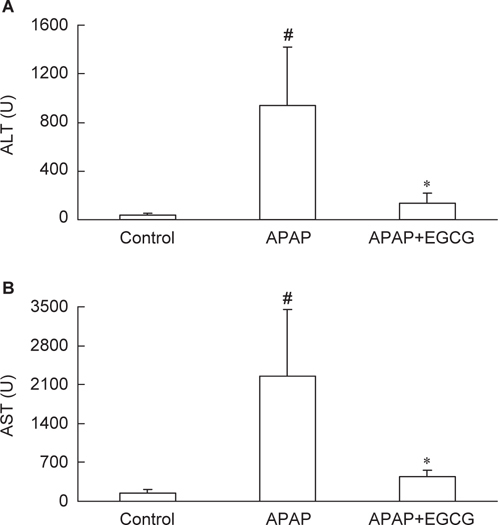
– Effect of EGCG feeding (0.54 %, w/w) on plasma alanine aminotransferase (ALT) (A) and aspartate aminotransferase (AST) (B) in rats after APAP treatment. #Significantly different from control *(P* < 0.05). * Significantly different from APAP (*P* < 0.05). Values are the mean± SD of n = 6.

After a single dose of the APAP treatment, there were no significant differences in body weight and liver weight among the three groups (data not shown). APAP treatment increased plasma ALT and AST activities compared with those same activities in control animals (*P* < 0.05) (Figure 1A, 1B). However, rats fed the EGCG diets had lower (P < 0.05) plasma ALT and AST activities after APAP treatment.

**Fig. 2:**
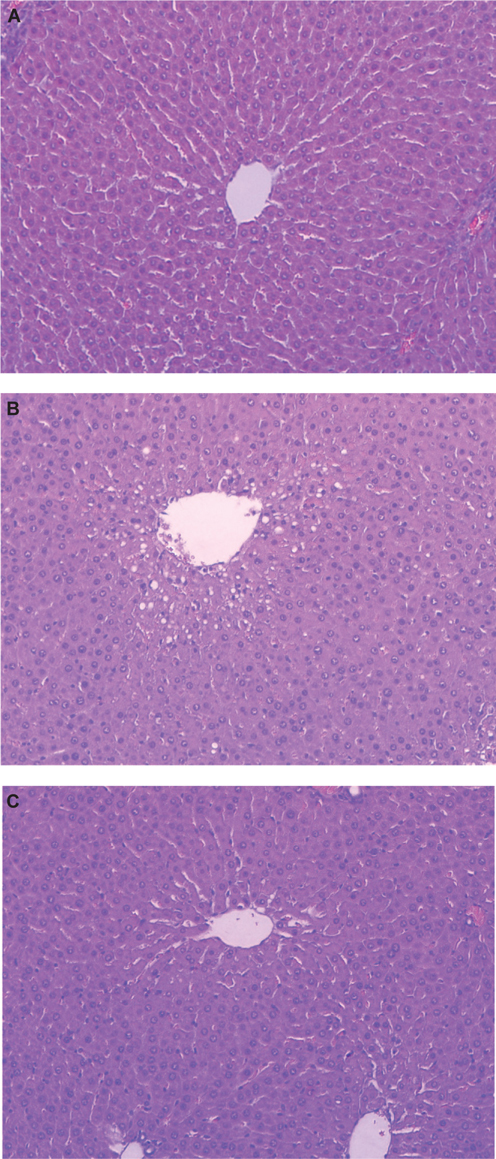
– Histological results of liver section stained with H&E under light microscope in rats (200×). (A), Control group; (B), APAP group; (C), APAP+EGCG group.

Histological examination of H&E stained liver sections was conducted 12 h after APAP administration to confirm the pattern of hepatotoxicity and compare the extent of liver injury between the control and the EGCG fed animals (Figure 2). Morphological findings were consistent with plasma transaminase observations. The APAP-induced histopathological changes in the liver came with significant degeneration and necrosis of hepatocytes in the centrilobular region and with perivenular inflammatory infiltrates. These APAP-induced histopathological changes were significantly reduced by EGCG treatment. These results indicate that the hepatotoxicity induced by APAP treatment in rats was reduced by EGCG.

After the intraperitoneal injection of a single dose of APAP, plasma APAP concentration remained unchanged in EGCG-treated rats (Table 2). However, EGCG feeding significantly reduced plasma APAP-glucuronide, APAP-sulfate, and APAP-glutathione concentrations in rat livers (*P* < 0.05). Lower *(P* < 0.05) APAP, APAP-glucuronide, and APAP-glutathione contents in the livers were noted in the EGCG group after APAP treatment. EGCG, however, had no effect on APAP-sulfate and APAP protein adducts contents in rat livers (*P* > 0.05).

**Table 2:** – APAP and its related conjugates in the plasma and the liver of the rats.

	APAP	APAP + EGCG
Plasma (us/ml)		
APAP	371.2± 126.2	387.5± 62.9
APAP-glucuronate	135.9± 10.0	83.5± 24.9*
APAP-sulfate	42.2± 3.4	36.9± 3.0*
APAP-glutathione	18.3± 2.2	10.7± 2.9*
Liver (us/s liver)		
APAP	116.4± 58.1	45.9± 18.6*
APAP-glucuronate	55.5± 3.5	32.2± 12.4*
APAP-sulfate	11.0± 0.8	10.3± 0.6
APAP-glutathione	58.2± 28.4	17.6± 4.1*
APAP protein adducts	40.5± 3.1	39.4± 2.7

Results are expressed as the mean± S.D. of six rats in each dietary group. The amount of EGCG in the diet was 0.54 % (w/w).

*Significantly different from APAP group, *P* < 0.05.

The effect of EGCG on drug-metabolizing enzymes after APAP treatment is shown in Table 3. After APAP treatment, there was no significant difference (*P* > 0.05) on the activities of methoxyresorufin O-demethylase (CYP1A2), nitrophenol 6-hydroxylase (CYP2E1), and midazolam 1-hydroxylation (CYP3A); however, GST, UGT, and ST activities were lower than in the control group (*P* < 0.05). Among the APAP-treated groups, CYP3A and CYP2E1 activities were significantly lowered (*P* < 0.05) by EGCG. A significant decrease (*P* < 0.05) in ST activity was observed in rats fed a diet containing EGCG. EGCG had no effect *(P* > 0.05) on UGT activity in rats treated with APAP. In addition, EGCG feeding reversed the reduction of GST activity that was induced by APAP (*P* < 0.05).

**Table 3:** – Effect of EGCG feeding on drug-metabolizing enzyme activities in rat livers.

	Control	APAP	APAP + EGCG
Midazolam 1-hydroxylation (CYP3A4) (pmol/min/mg protein)	213.1± 26.4	181.8± 29.0	126.2± 26.5*
Nitrophenol 6-hydroxylase (CYP2E1) (pmol/min/mg protein)	395.1± 83.5	318.5± 48.5	221.7± 81.2*
Methoxyresorufin O-demethylase (CYP1A2) (pmol/min/mg protein)	47.5± 9.4	45.6± 5.8	46.1± 4.1
UDP-glucurosyltransferase (nmol/min/mg protein)	36.2± 3.4	30.4± 2.3#	28.6± 5.5
Sulfotransferase (pmol/min/mg protein)	339.8± 57.0	223.0± 56.8#	137.9± 43.7*
Glutathione S-transferase (nmol/min/mg protein)	192.9± 5.3	177.9± 8.0#	192.2± 9.8*

Results are expressed as the mean± S.D. of six rats in each dietary group. The amount of EGCG in the diet was 0.54 % (w/w).

# Significantly different from control group; *Significantly different from APAP group, *P* < 0.05.

**Fig. 3:**
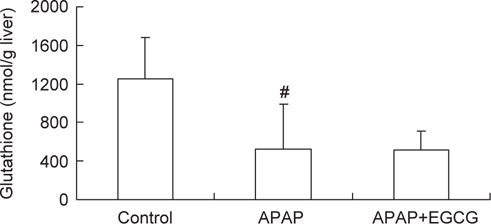
– Effect of EGCG feeding (0.54 %, w/w) on GSH levels in rats after APAP treatment. #Significantly different from control (*P* < 0.05). Values are the mean± SD of n = 6.

A dramatic decrease (*P* < 0.05) in the hepatic GSH level was found in the APAP-treated groups. EGCG had no significant effect (*P* > 0.05) on hepatic GSH content after APAP treatment (Figure 3).

## 4. Discussion

The results of the present study show that EGCG feeding significantly reduced the elevation of plasma ALT and AST activities that were first induced by APAP. Morphological damage by APAP was lower in rats fed the EGCG diet. In addition, EGCG feeding reduced CYP3A and CYP2E1 activities and lowered APAP-glutathione content in rat livers. These results suggest that EGCG feeding may reduce CYP-mediated APAP bioactivation in liver and, at least in part, contribute to its ability to lower hepatotoxicity.

In addition to lower plasma ALT and AST activities, we also observed that morphological damage by APAP was lower in rats fed the EGCG diet. In this study, hepatic CYP2E1 and CYP3A activities after APAP treatment were reduced in rats fed the EGCG diet. CYP3A and CYP2E1 are two major enzymes that catalyze the oxidative metabolism of APAP and that may generate the toxic electrophile NAPQI [25]. Higher CYP3A and CYP2E1 activity may enhance APAP toxicity by increasing CYP-mediated electrophilic NAPQI formation [26, 27]. Therefore, the lower CYP3A and CYP2E1 activities after APAP treatment in the livers of rats fed the EGCG diet may reduce NAPQI formation and thus lower the formation of conjugates with GSH. This would result in lower APAP-glutathione formation in the liver (Table 2). Recently, APAP-glutathione was shown to be toxic to the liver because the conjugate can induce mitochondrial impairment, leading to enhanced reactive oxygen species production [28]. These results suggest that EGCG could reduce APAP-induced hepatotoxicity, an effect which might be partially attributed to the lower APAP-glutathione levels in the liver after treatment with EGCG (Table 2).

In addition, it has been reported that the formation of the APAP protein adducts level in the liver after APAP overdose displays a high incidence of hepatotoxicity [29]. It was surprising and notable that EGCG had no effect on the APAP protein adducts level in the rat livers even though it could lower hepatic CYP enzyme activities and APAP-glutathione formation. The reason for this observation is unknown in this study and further investigation is warranted to clarify the other mechanism(s) of EGCG on reducing hepatotoxicity.

In addition to lower APAP-glutathione, it was noteworthy that rats fed the EGCG diet also reduced APAP (-60.6 %) and APAP-glucuronate (-42 %), the major metabolites of APAP, in their livers. In our preliminary study, EGCG significantly reduced not only CYP3A but also UGT, SUT, and GST activities in rat livers (Table 1), and those of enzymes are responsible for the metabolism of APAP. These results suggest that the APAP metabolism in the liver was reduced by EGCG. The lower UGT activity might partially explain the lower APAP-glucuronate content in the livers (Table 2). In addition, rats fed tea polyphenols have been shown to have reduced microbial Β-glucuronidase activity in the cecum [30]. Therefore, it is possible that EGCG feeding may have reduced enzymatic deconjugation of APAP-glucuronate in the intestine and decreased the re-absorption of APAP *via* the enterohepatic circulation pathway. The lowered re-absorption of APAP may result in the lower *(P* < 0.05) APAP content in the liver and thus reduce the CYP-mediated bioactivation of APAP.

In summary, EGCG may act as a hepatoprotective agent against APAP-induced liver injury. Although the exact mechanism is still not clear, our study is the first to demonstrate that feeding rats a diet containing EGCG for 1 week reduces the metabolism and toxicity of APAP

## Acknowledgments

This study was financially supported by the grant-aid (NSC 102-2313-B-039-007) of the National Science Council, Taiwan.
